# Rapid and Label-Free Separation of Burkitt's Lymphoma Cells from Red Blood Cells by Optically-Induced Electrokinetics

**DOI:** 10.1371/journal.pone.0090827

**Published:** 2014-03-07

**Authors:** Wenfeng Liang, Yuliang Zhao, Lianqing Liu, Yuechao Wang, Zaili Dong, Wen Jung Li, Gwo-Bin Lee, Xiubin Xiao, Weijing Zhang

**Affiliations:** 1 State Key Laboratory of Robotics, Shenyang Institute of Automation, Chinese Academy of Sciences, Shenyang, China; 2 University of Chinese Academy of Sciences, Beijing, China; 3 Department of Mechanical and Biomedical Engineering, City University of Hong Kong, Kowloon, Hong Kong; 4 Department of Power Mechanical Engineering, National Tsing Hua University, Hsinchu, Taiwan; 5 Department of Lymphoma, Affiliated Hospital of Military Medical Academy of Sciences, Beijing, China; Institute for Frontier Medical Sciences, Kyoto University, Japan

## Abstract

Early stage detection of lymphoma cells is invaluable for providing reliable prognosis to patients. However, the purity of lymphoma cells in extracted samples from human patients' marrow is typically low. To address this issue, we report here our work on using *optically-induced dielectrophoresis* (ODEP) force to rapidly purify Raji cells' (a type of Burkitt's lymphoma cell) sample from red blood cells (RBCs) with a label-free process. This method utilizes dynamically moving virtual electrodes to induce negative ODEP force of varying magnitudes on the Raji cells and RBCs in an *optically-induced electrokinetics* (OEK) chip. Polarization models for the two types of cells that reflect their discriminate electrical properties were established. Then, the cells' differential velocities caused by a specific ODEP force field were obtained by a finite element simulation model, thereby established the theoretical basis that the two types of cells could be separated using an ODEP force field. To ensure that the ODEP force dominated the separation process, a comparison of the ODEP force with other significant electrokinetics forces was conducted using numerical results. Furthermore, the performance of the ODEP-based approach for separating Raji cells from RBCs was experimentally investigated. The results showed that these two types of cells, with different concentration ratios, could be separated rapidly using externally-applied electrical field at a driven frequency of 50 kHz at 20 V_pp_. In addition, we have found that in order to facilitate ODEP-based cell separation, Raji cells' adhesion to the OEK chip's substrate should be minimized. This paper also presents our experimental results of finding the appropriate bovine serum albumin concentration in an isotonic solution to reduce cell adhesion, while maintaining suitable medium conductivity for electrokinetics-based cell separation. In short, we have demonstrated that OEK technology could be a promising tool for efficient and effective purification of Raji cells from RBCs.

## Introduction

B-cell lymphomas are a species of lymphomas derived from the carcinogenesis of B lymphocytes in the human lymphatic system. They are generally classified into two categories: 1) indolent lymphomas – cancerous cells that are under control and patients have a long-term survival rate even without treatments; and 2) malignant lymphomas – which are cancerous cells that could spread rapidly and cause a rapid deterioration of the health and even death of patients, and hence, need timely and thorough treatments. Burkitt's lymphoma [1], one of the fourteen kinds of B-cell lymphomas, is a type of malignant lymphoma and propagates quickly inside a patient's body, often to the bone marrow, blood, and central nervous system. Without timely treatment, Burkitt's lymphoma could cause death rapidly. However, this kind of malignant lymphoma can be cured, depending on the histology, type, and stage of the disease [2]. Thus, early stage detection of this type of lymphoma cell is essential and invaluable for achieving a favorable prognosis, as well as for potentially improving the patient's quality of life. However, different patients may exhibit varying degrees of drug resistance to the same drugs commonly used in targeted therapy for the clinical treatment of lymphomas. Thus, it is necessary to explore the clinicopathological characteristics of these cancerous cells from human lymphoma patients in order to better understand the relationship between cell histology and disease pathology in patients. Correlating data of cell histology and disease pathology to improve the accuracy of an early patient diagnosis will assist doctors in choosing the best treatments for patients. However, there are typically many red blood cells (RBCs) in a solution sample of Raji cells (a type of Burkitt's lymphoma cell) extracted from patients. Thus, a rapid and efficient technique is required to enable the identifying, discriminating, and purifying of target Raji cells in a mixed cell population from RBCs that may interfere with later detection and research protocols. For this purpose, technologies with a high degree of sensitivity, specificity, and reproducibility are required to separate Raji cells from RBCs. Existing technologies are broadly categorized using specific biological markers or differential biomechanical and electromechanical schemes. Of these schemes, biomechanical and electromechanical methods are known as “label-free” techniques as no biomarkers are required to implement them. For example, the density gradient centrifugation method [3]–[4] is a label-free method commonly used to remove the RBCs or plasma for isolating the cancerous cells in peripheral blood, using the density variation mechanism of cells with the assistance of commercial available liquid kits (e.g., using Ficoll as given in [5]). This technique, however, simultaneously contaminates all of the isolated RBCs. Another label-free technique is using microfluidic systems, i.e., based on purely hydrodynamic forces. This technique has already been demonstrated to be capable of isolating cancerous cells with a recovery rate of over 90% [6]. However, a strong drawback of this method is that separation of cells of similar inertia (i.e., similar sizes) is very difficult. *Acoustophoresis* is another separation method based on biomechanical mechanism, and has been employed to separate cancer cell lines from leukocyte fractions by acoustic standing-wave forces with a purity of over 79.6% [7]. Nevertheless, acoustophoresis seems to be a low throughput method, since separation performance is determined by retention time in the acoustic field (i.e., slow flow rate in the microfluidic channel is desired), the size of the cells, and the acoustic properties of the cells relative to the suspending medium. Alternatively, *immunomagnetic-based cell separation* approaches are commonly used to identify and discriminate cancerous cells by coupling magnetic beads with cell surface antigen-specific antibodies with an auxiliary of applied magnetic field [8]–[9]. This approach is based on the identification of selected proteins on the tumor cells' surface, including epithelial cell adhesion molecule, cytokeratins, and other proteins. However, these proteins are not expressed in all tumors [10] and, hence, some cells cannot be labeled and targeted, which limits the applications of this technique for cell separation. Moreover, the preparation of cell samples is also more complicated and time-consuming than other methods. In addition, dielectrophoresis (DEP) force, generated by the interaction between an applied non-uniform electric field and the dielectric objects in the field, can be used to induce motions of different directions on the objects, reflecting the differential inherent properties (e.g., dielectric properties and sizes) of the objects. Accordingly, this technique is a label-free manner and has been applied to separate cancerous cells [11]–[13]. Nevertheless, the DEP-based separation technique requires unique conductive electrodes fabricated by micro-lithographic techniques to be integrated with a microfluidic system, and, hence, this technology lacks the flexibility of allowing for dynamic and reconfigurable manipulation once the electrodes are fabricated. Moreover, integrated micro pumps/valves are needed to enhance the separation performance, which complicates the fabrication process of the experimental system.

More recently, *optically-induced dielectrophoresis* (ODEP), or *optical electronic tweezer* (OET) [14], similar in its operational principle to DEP, permits researchers to dynamically alter the virtual electrodes that serve the function of generating the DEP force in a real-time, programmable, and reconfigurable approach by controlling the optically-projected patterns that are digitally generated by a computer. This relatively new ODEP force-based micro-/nano-manipulation technique has already been demonstrated in a large variety of applications in the bioengineering field, including cell separation (separation of live and dead human B-cells) [15], discrimination of normal oocytes [16], manipulation of DNA molecules [17], cell counting and cell lysis [18], sperm diagnostic manipulation [19], mouse embryo selection [20], circulating tumor cell isolation [21]–[22], and cancerous cell identification [23]. We present in this paper our experimental validation of rapidly separating Raji cells from RBCs by employing a *dynamic optically-induced DEP force* in an OEK (*optically-induced electrokinetics*) chip. The polarization models for the two types of cells were established, and then the finite element method (FEM) numerical solutions of their velocities induced by the ODEP force were obtained with the purpose of demonstrating the feasibility of using two optically-projected lines (with one dynamically moving and one stationary) of different widths to separate the two types of cells. We note that, in addition to the ODEP force, there could simultaneously exist two other types of electrokinetics forces, namely AC electroosmosis (ACEO) and AC electrothermal (ACET) flows, during the cell manipulation and separation process. These two forces, ACEO and ACET, are due to the interaction of the electric double layers with the tangential component of the electric field and the presence of the non-uniform electric field resulting in Joule heating, respectively. Consequently, we herein define the microfluidic device used in our experiments as an “*optically-induced electrokinetics*” (OEK) chip, instead of an “ODEP chip” or and “OET chip”. Moreover, within an applied electric field across the OEK device, Raji cells tended to randomly adhere to the a-Si:H surface and, thus, influence cell separation performance. To reduce this cell-adhesion phenomenon, the composition of the isotonic solution in which the two types of cells are suspended was experimentally optimized and the results are reported there. With a proper percentage of bovine serum albumin (BSA) in an isotonic solution, the affinity force between the cells and the bottom substrate surface of the OEK chip (i.e., the a-Si:H layer) could be decreased significantly, and, hence, enhance the success rate of the ODEP-based manipulation and separation of cells. Most importantly, the separation of Raji cells and RBCs with two different concentration ratios were experimentally explored using an OEK chip, and the results are presented in this paper.

## Theory and FEM modeling

### Generation of “ODEP” force

**Figure 1:**
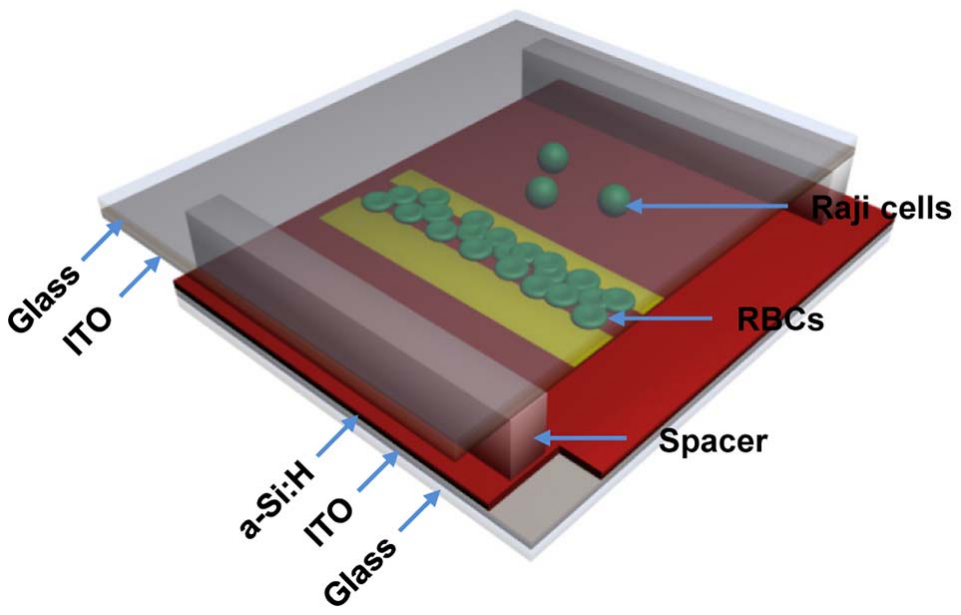
Three-dimensional schematic illustration of the OEK chip.

Figure 1 is a diagrammatic sketch of our OEK chip, which consists of a top glass substrate coated with a transparent and conductive indium tin oxide (ITO) film that is used as an electrode; a microfluidic chamber that is the working area of the chip (the volume of this chamber is 3 cm ×1 cm ×60 μm); and a bottom substrate with a thin photoconductive film of hydrogenated amorphous silicon (a-Si:H) deposited onto another ITO glass substrate. The a-Si:H layer behaves as an insulator due to its inherent lower conductivity when not illuminated. When an optical pattern is projected onto a particular area of the a-Si:H layer via a commercial digital projector, the electron-hole pairs are excited by the migration of electrons from the valence band to the conduction band of this corresponding area of the a-Si:H layer and, thus, locally increases the conductivity of the illuminated area of the a-Si:H layer via the photoconductive effect. Then, the electric field across the liquid chamber dramatically increases above the locally illuminated a-Si:H area since most of the externally applied voltage is shifted to the liquid chamber; thus, a non-uniform electric field can be created in the liquid chamber. Additionally, any dielectric particles suspended in the liquid at locations near this optically-induced non-uniform electric field will experience a force caused by an interaction between the electrically-polarized dipole moments of both of the particles and the liquid solution, known as the “ODEP force” in this OEK chip. Unlike conventional DEP chips, no metal electrodes are required to create the non-uniform electric field; hence, ODEP provides a dynamic and flexible manipulation technique. The ODEP force can be either positive or negative under specific conditions, which means that the particles can be either attracted to (pDEP), or repelled from (nDEP), the illuminated areas, respectively. Our prior work presented in [24] provides a more detailed treatment of how optical wave-lengths, applied AC wave forms, and frequency can affect the optically-induced DEP force.

### Polarization models for Raji cells and RBCs

**Figure 2:**
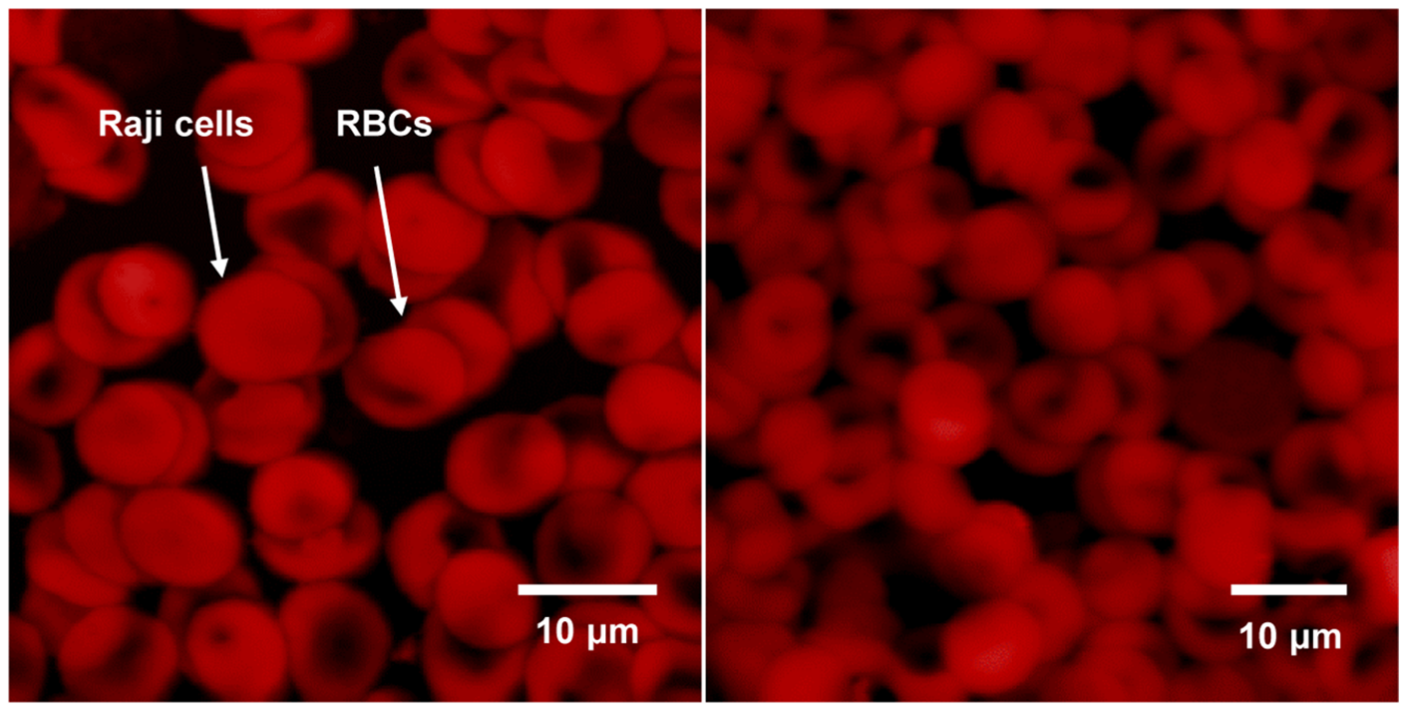
Atomic force microscope-scanned images of Raji cells and RBCs.

The time-averaged DEP force exerted on a dielectric particle in a fluidic medium is given as [25]:
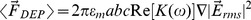
(1)where *a, b,* and *c* are the semi-axes of the particle radii, respectively; *ε_m_* denotes the permittivity of the liquid medium; *E_rms_* is the root-mean-square value of the electric field; and Re[*K*(*ω*)] is the real part of the Clausius-Mossotti (CM) factor, which is further expressed as [25]:



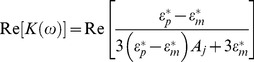
(2)In the above equation, ε^*^  =  ε – jσ/ω, where *ε* and *σ* are the permittivity and conductivity, respectively; *ω* = 2π*f*, where *f* is the applied voltage frequency across the liquid medium; the subscripts *p* and *m* denote the properties of the particle and liquid medium, respectively; and *A_j_* is the depolarization factor along the *j*-axis (*j*  =  *x*, *y*, *z*). Intuitively, based on Eq. (1), the DEP force depends on a particle's geometry and inherent dielectric property. We therefore speculated that the separation of Raji cells and RBCs is feasible because these cells have very different geometric shapes and dielectric properties. Figure 2 shows atomic force microscope (AFM) scanned images of both Raji cells and RBCs, indicating their large differences in shape. The remaining parts of this paper will show, both theoretically and experimentally, that our intuitive conjuncture was correct.

#### a) Raji cells

**Figure 3:**
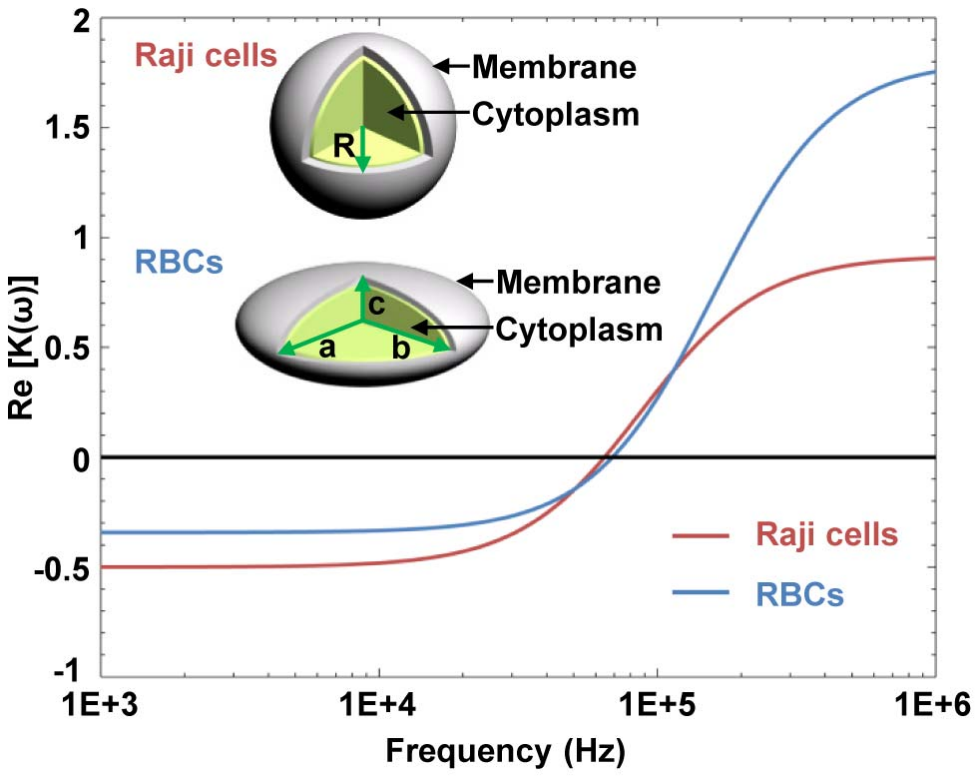
Re[K(ω)] for the two types of cells as a function of the applied frequency. The two types of cells are suspended in an isotonic solution with a conductivity of 1.3×10^−2^ S/m. Positive values represent an attractive (positive) DEP force, and negative values represent a repulsive (negative) DEP force.

Raji cells can be modeled as a spherical particle (*R*  =  *a*  =  *b*  =  *c*, *A_j_*  = 1/3), as shown in the inset of Figure 3. The shell and interior refer to the cell membrane and to the cytoplasm, respectively. Consequently, a single layer of the shell-core model can be used as an approximation for Raji cells, and the effective complex permittivity for Raji cells is given by [26]:
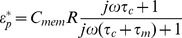
(3)where *τ_c_*  =  *ε_c_ /σ_c_*, *τ_m_*  =  *C_mem_R/σ_c_*.; the subscripts *c* and *mem* represent the cell cytoplasm and membrane, respectively; *C_mem_* is the capacitance of the cell membrane; and *R* is radius of the cell. The real part of the CM factor for Raji cells is further given by [26]:
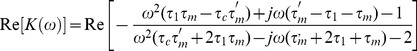
(4)where *τ_1_*  = *ε_m_*/*σ_m_*, *τ_m_*' =  *C_mem_R/σ_m_*.

#### b) RBCs

RBCs are modeled as single-shell oblate ellipsoids having semi-axes of *a*, *b*, and *c*, which satisfy the relationships where *a*  =  *b* > *c* and *e*  = *a/c*  = 4, as shown in the inset of Figure 3. The shell and interior also refer to the cell membrane and the cytoplasm, respectively. The effective complex permittivity for RBCs is given by [27]:
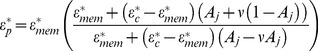
(5)where *ν* is the volume fraction of the cell interior; *ν*  =  *(c-d)(a-d)^2^/ca^2^*; and *A_j_* is the depolarization factor along the long axis, *A_j_*  = 0.5(*e* ^2^arctan(*e* ^2^−1)^0.5^−(*e* ^2^−1)^0.5^)/(*e* ^2^−1)^1.5^ [27]. The polarization properties for Raji cells and RBCs, i.e., the real part of their respective CM factors, as a function of the applied frequency, can be obtained by using (2), (4), and (5), as shown in Figure 3. Herein, the negative value for each of the two curves in Figure 3 represents a nDEP force under the given frequency range; therefore, a pDEP force will be exerted on the cells with an applied frequency higher than the crossover frequency. Typical dielectric parameters for the two types of cells, as shown in Table 1, are used to calculate the polarization for each cell type, which is suspended in an isotonic solution with a conductivity of 1.3×10^−2^ S/m.

**Table 1:** Dielectric properties of Raji cells and RBCs.

Type	*σ_c_*(S/m)	*ε_c_*(F/m)	*σ_mem_*(S/m)	*ε_mem_*(F/m)	*C_mem_*(F/m^2^)	*D *(nm)	R (μm)
Raji [28]	0.58	60ε_0_	8.2e-6	8.8ε_0_	1.07e-2	7	6
RBCs [29]	0.5	60ε_0_	1.6e-6	8.5ε_0_	8.7e-3	8	a = 3.5

### Raji cells and RBCs velocities induced by the ODEP force

**Figure 4:**
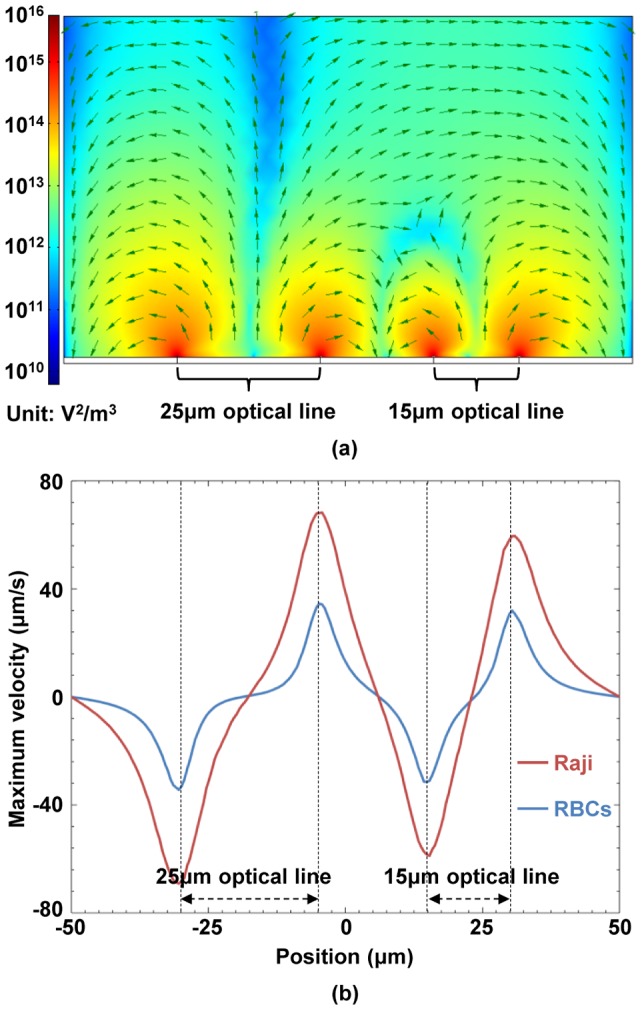
FEM simulation results of the DEP force. (a) Cross-sectional view of the x-component of the vector 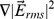. The arrows indicate the direction of the nDEP force induced by two optical lines of different widths, i.e., one moving line with a width of 25 μm and the other line being stationary with a width of 15 μm. (b) Velocities of Raji cells and RBCs induced by the ODEP force. The velocity of the Raji cells caused by the DEP force is higher than that of the RBCs, and the DEP force induced by the 25 μm-wide optical line for cells is also higher than that by the 15 μm optical line for cells.



Once a cell begins to move due to the DEP force, an opposing Stokes' drag force will be produced to retard the cell's motion. The Stokes' equation for estimating the drag force on a cell moving in a fluid with velocity *υ* is given as [30]

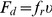
(6)where *f_r_* is the friction factor of the cell in the fluid. Considering the geometrical configurations of the two types of cells, the friction factor for Raji cells and RBCs are 6π*ηR* and 32/(3*ηa*) [25], respectively, where *η* is the dynamic viscosity of the fluid. Since the Stokes' drag force is directly proportional to the particle's moving velocity, the magnitude of the DEP force acting on a particle can be experimentally inferred. However, in order to theoretically obtain the magnitude of the DEP force exerted on the two types of cells, the vector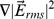 in (1) should be calculated. As an example, Figure 4(a) shows the cross-sectional distribution of the x-component of the vector 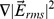
(denoted as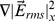) calculated using a commercial FEM software package (Multiphysics, COMSOL AB, Sweden). The FEM simulation method is the same as discussed in our prior work [31]. In this example, two optically-projected lines with a width of 25 μm and 15 μm, respectively, serve as virtual electrodes and the applied frequency is 50 kHz with a voltage of 20 V_pp_. These virtual electrode geometries and electric field parameters reflect the actual experimental conditions used in our Raji cells and RBCs separation experiments, as will be discussed in the ‘Results and discussions’ section. As shown in Figure 4(a), the DEP force sharply decreases along the vertical direction and has a maximum value around the illuminated areas. As illustrated and described in our prior work [24], the maximum DEP force exerted on the two types of cells can be determined at the position of the height of cells' radii above the bottom of the a-Si layer, defined as

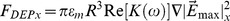
(7)Then, the velocity for Raji cells and RBCs induced by the maximum DEP force can be obtained by balancing (6) and (7), expressed as
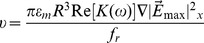
(8)


As shown in Figure 4(b), the maximum velocity of the Raji cells, as induced by the DEP force, is higher than that of the RBCs; additionally, the DEP force induced by the 25 μm-wide optical line is higher than that induced by the 15 μm optical line. This means that the DEP force induced by two different optical line widths can be employed to perform the separation of Raji cells from RBCs.

### Comparison of AC- and optically-related electrokinetics forces

**Figure 5:**
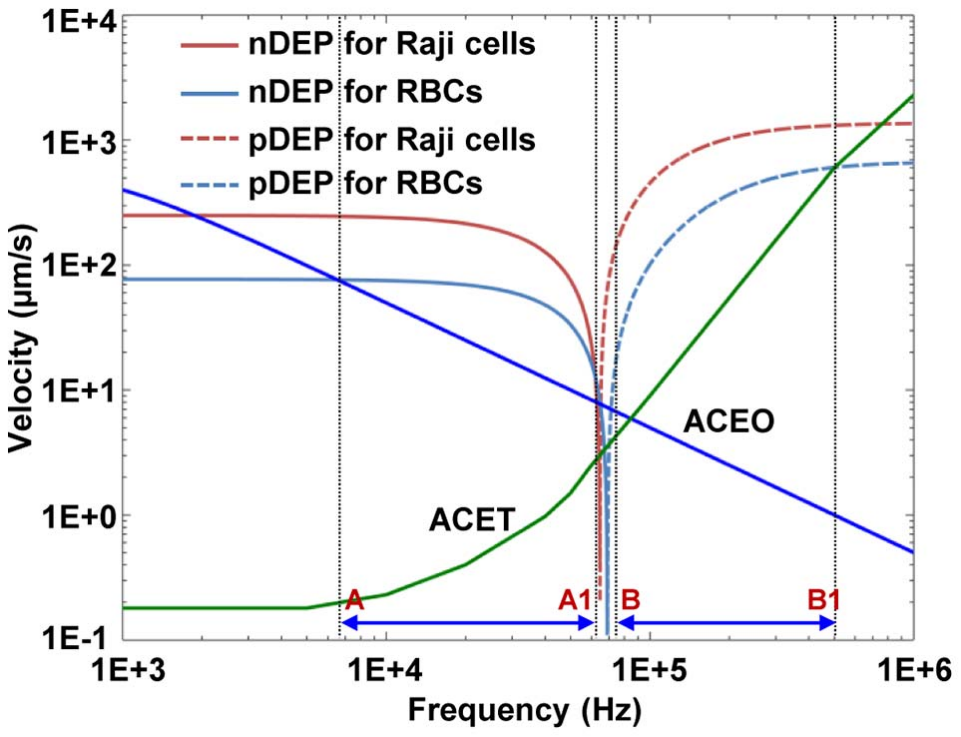
Contribution of various optically-induced electrokinetics forces on the velocities for Raji cells and RBCs as a function of the applied frequency. The velocity generated by the DEP, ACEO, and ACET were at 6 μm and 3.5 μm height above the a-Si:H film surface for Raji cells and RBCs, respectively. The width of the optical line that served as the virtual electrode to generate the electric field was 25 μm.

As noted in the previous section, there exist several optically-related electrokinetic forces in an OEK chip, mainly including the DEP force and forces on a particle caused by ACEO- and ACET-induced flows. To enhance the experimental performance of the separation of the two types of cells, experimental conditions should be set up such that the DEP force will be dominant in the separation process with a specific applied voltage frequency across the upper and lower substrates of an OEK chip. Using the equations in Table 2, the numerical solution showing the velocities for the two types of cells caused by the AC electrokinetics forces (i.e., DEP, ACEO, and ACET), as a function of the applied frequency, were obtained by using the FEM method with same simulation parameters and simulation modules as those described in [31], except that a liquid conductivity of 1.3×10^−2^ S/m was used to reflect the experimental conditions for the work reported in this paper. Figure 5 shows the simulated and calculated results of the cellular velocities induced by various forces. The results indicate that within specific frequency range, the ACEO or ACET may affect the DEP force-based separation process, respectively. Furthermore, for these two types of cells, when the frequency is from ∼7 kHz to ∼60 kHz, the nDEP force will be the dominate force; when the frequency is from ∼70 kHz to ∼500 kHz, the pDEP force will be the dominant force on both the RBCs and the Raji cells. However, the ACET force in the frequency range of ∼70 kHz to ∼500 kHz is much higher than that in the frequency range of ∼7 kHz to ∼60 kHz, and thus may affect the cellular properties due to the increased temperature in the fluidic medium. Hence, a frequency in the range of ∼7 kHz to ∼60 kHz, which induces an nDEP force, is selected to conduct the separation experiments in this study.

**Table 2:** Equations of the velocities caused by the optically-induced electrokinetics forces exerted on the two types of cells.

Forces	Governing Equations [30]
DEP	*Equation 8*
ACEO	-*ε*_m_ 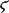 *E*_t_/*η*
ET flow	0.5*ε*_m_[(*α*-*β*)(▿*T*·*E*)*E* ^*^/(1+(*ωτ*)^2^)- 0.5*α*|*E*|^2^▿*T*]

Here, 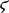 is the zeta potential; *η* is the dynamic viscosity of DI water; *E*_t_ is the tangential electric field; *T* is the temperature of the liquid solution; and *τ*  =  *ε*_m_/*σ*_m_. For DI water at room temperature, approximately, *k* = 0.6 J·m^−1^ s^−1^·K^−1^; *ρ*_m_  = 1 g·cm^−3^, *α = *−0.4% K^−1^; *β* = 2% K^−1^; and ∂*ρ*_m_/∂*T/ρ*_m_  = 10^−4^ K^−1^ [30].

## Materials and Methods

### Fabrication of the OEK chip

The fabrication process of the OEK chip employed in this paper was described in detail in our prior work [32]. In order to apply an electric field across the chip, part of the a-Si:H thin film on the bottom ITO-glass substrate was etched to establish an electrical connection, as shown in Figure 1. Specifically, a 5 mm ×8 mm area of a-Si:H was patterned through standard photolithography and dry-etching (using the Oxford Plasma Lab 80 etching system) with 2% oxygen, 12.5% CF4 gas, with a 30mTorr etching chamber pressure, and 6-minute plasma exposure. The chip was then rinsed and cleaned with acetone and DI water before being dried by nitrogen gas. The microfluidic chamber, into which the cells and solution were injected, has a height of ∼60 μm. This microfluidic chamber between two ITO-glass substrates was constructed by using a patterned polydimethylsiloxane (PDMS) thick film or a double-sided tape as a spacer.

### Experimental setup

**Figure 6:**
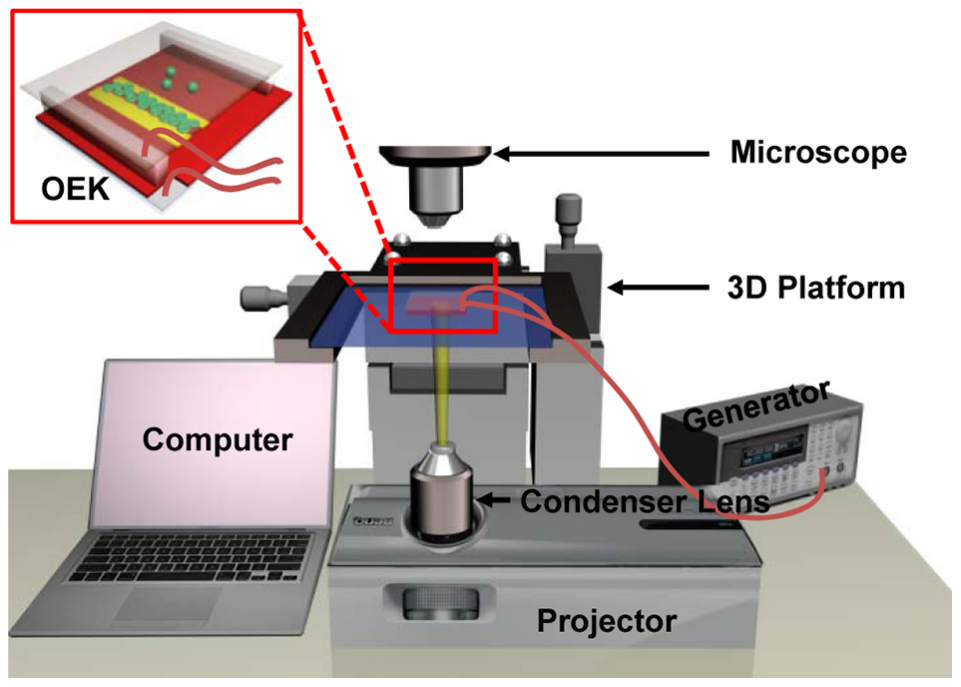
Schematic illustration of the experimental setup for the OEK chip. The experimental system consists of an image acquisition system to observe the cell separation process, and a pattern generation system to generate the virtual electrodes which are projected onto the lower surface of the OEK chip by a LCD projector.

The experimental setup for our OEK platform is shown in Figure 6 (illustration) and Figure S1 (picture). The OEK chip was fixed on a three-dimensional digital translation platform (Leetro Automation Co. Ltd, China), which would accurately regulate the spatial movement of the OEK chip, as well as span the working areas on the chip. To create the optically-projected patterns, a commercial graphics software package (Flash 11, Adobe, U.S.A.) was employed to generate the virtual electrodes with any desired geometrical configurations, which were projected onto the lower surface of the OEK chip via a commercial LCD projector (VPL-F400X, Sony, Japan) coupled with a computer. The manipulation and separation processes of the cells were observed and recorded using a charged coupled device (DH-SV1411FC, DaHeng Image, China) mounted on a microscope (Zoom 160, OPTEM, U.S.A.). In addition, a condenser lens (Nikon, MS plan, 50×), fixed between the LCD projector and the OEK chip, was used to focus and collimate the optical pattern onto the OEK chip. In order to power the OEK chip to perform cell separation process, an AC bias potential, supplied by a function generator (Agilent 33522A, U.S.A.), was applied to the transparent ITO glasses, which are located at the top and bottom of the OEK chip as shown in Figure 1.

### Cell preparation and counting

The RBCs were obtained from volunteers as previously described in [33]. The samples were centrifuged at 1000 rpm for 5 min at 4°C (Sigma 3–30K, Germany). Then, the supernatant was discarded and the remained RBCs were resuspended into 1 mL of the isotonic solution for a second round of centrifugation using the same parameters. Then, the collected RBCs were resuspended into 1mL of isotonic solution for further experiments. The purpose of using the isotonic solution was to maintain an appropriate osmotic condition for the viability of the cells.

The Raji cell line [34] was a gift from Dr. Xiubin Xiao of the Affiliated Hospital of Military Medical Academy of Sciences, Beijing, China. The Raji cell line was cultured in Roswell Park Memorial Institute (RPMI-1640) culture medium supplemented with 10% (v/v) fetal calf serum, 1% penicillin (v/v) (100 U/mL), and 1% streptomycin (v/v) (100 μg/mL) at 37°C in a humidified atmosphere of 5% CO_2_ (Model 371, Thermo Scientific). Before each experiment, 1 mL of Raji cell suspension was taken directly out of the culturing flask, and centrifuged at 1000 rpm for 5 min at 4°C with supernatant discarded. The collected Raji cells were resuspended into 1 mL of RPMI-1640 medium and centrifuged again using the same parameters to remove the residual culture medium. Then, the resulting Raji cells were resuspended into 1 mL of isotonic solution for further experiments.

After the cell suspensions were prepared, cells counts were performed for both types using a commercial hemocytometer (Qiujing Co. Ltd., China) to control the cell concentration. In order to achieve standardized and comparable cell separation results using the proposed OEK chips, 1 mL of Raji cells suspension with a concentration of 1×10^6^ cells/mL was spiked into 1 mL of RBCs suspension to obtain various cell ratios. For our experiments, RBCs concentrations of 1×10^7^ cells/mL and 4×10^7^ cells/mL were used to achieve cells ratios (Raji cells: RBCs) of 1∶10 and 1∶40, respectively. Finally, the resulting cellular mixture of RBCs and Raji Cells was loaded into the OEK chip for further ODEP force based cell separation experiments.

## Results and Discussion

### Investigation of Raji cellular adhesion phenomenon inside an OEK chip

**Figure 7:**
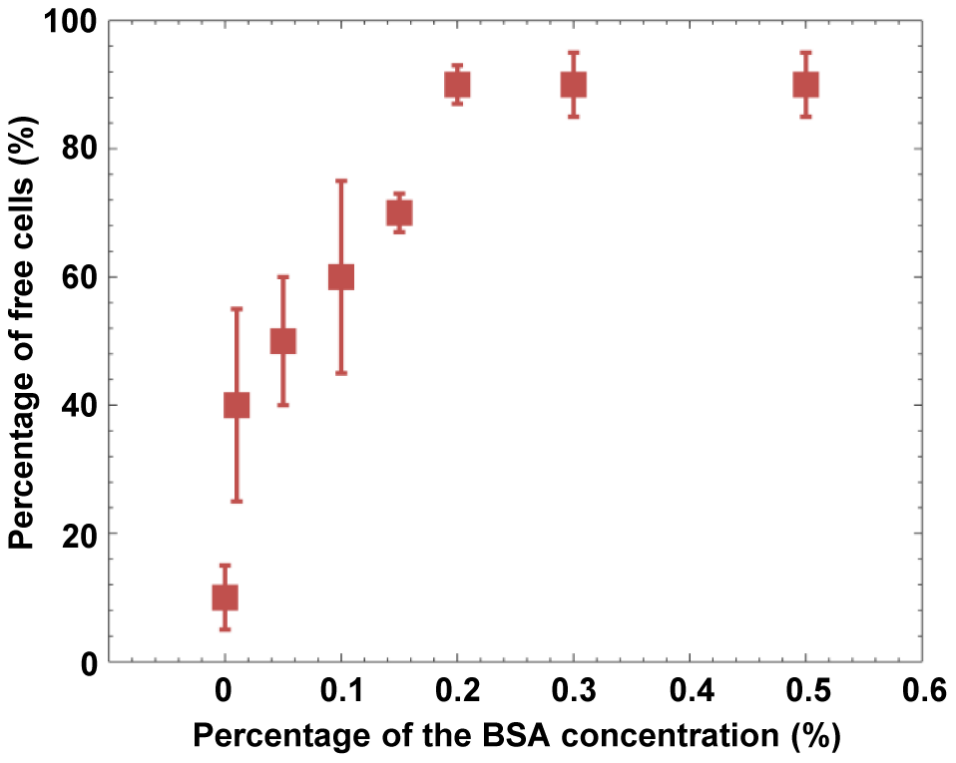
Percentage of free cells as a function of the percentage of the BSA concentration. Raji cells to substrate adhesion could be significantly decreased when 0.2% BSA was added to the conventional isotonic solution. The error bars indicate the standard deviation of the measurements.

In a typical experimental procedure to investigate the cell-to-substrate adhesion phenomenon, Raji cells (at a concentration of 5×10^5^ cells/mL) suspended in an isotonic solution consisting of 8.5% (w/v) sucrose and 0.3% (w/v) glucose were introduced into the OEK chip (with a microfluidic chamber that holds ∼18 μL solution). All Raji cells within the microscope's field of view (280 μm ×200 μm) were then investigated. For the purpose of data analysis, the cells that could be moved by the optically projected patterns (i.e., virtual electrodes) are defined as “free cells”. And, the cells that did not show any movement when ODEP force was applied around them are defined as “adherent cells”. For a typical experiment, ∼90 cells in the field of view of the microscope were investigated. In addition, these tests were performed in ten different OEK chips. Moreover, ten different “field of view areas” (FOVA) were investigated in each chip. Two experiments were performed for each of the FOVA in each of the ten OEK chips used in this study. Accordingly, 200 experiments carried out. Therefore, we have observed the adhesion behavior of ∼18, 000 cells (90 cells ×2×10 FOVA ×10 OEK chips) in obtaining each data point shown in Figure 7. The experimental results showed that only ∼10% “free cells” of the Raji cells could be freely transported by ODEP force if only the isotonic solution is used, since the a-Si:H has a native oxide present at the a-Si:H/liquid interface, as described by N. K. Lau, et al., [35]. According to them, the adhesion force between a-Si:H surface and mammalian cells were in the order of nanonewtons, while the ODEP force was in the order ranging from tens to hundreds of piconewtons. Consequently, in order to facilitate ODEP-based cell separation, this adhesion force between the Raji cells and the a-Si:H surface should be minimized. N. K. Lau, et al., have reported chip-modification methods that can avoid cell adhesion by surface treatments; however, the processes for fabricating those modified chips are time-consuming and complicated. In this study, the method of adding different concentrations of bovine serum albumin (BSA) into the isotonic solution was experimentally investigated and characterized. The BSA was added into the solution with the purpose of decreasing the affinity force between the cells and the a-Si:H substrate of the OEK chip, and thereby enhancing the performance of the ODEP manipulation and separation process of the two types of cells. We have investigated cell-to-substrate adhesion behavior as a function of the BSA's concentration in the isotonic solution. As mentioned above, for each of the BSA concentration tested, Raji cells were observed in ten distinct field of views of the microscope for each of the ten different OEK chips. Results shown in Figure 7 revealed that the cell-to-substrate adhesion could be quite significantly decreased, i.e., with 90±3% of the cells being easily transported when 0.2% (wt) BSA was added to the conventional isotonic solution. Furthermore, the measured liquid conductivity of the optimized isotonic solution was 1.3×10^−2^ S/m (obtained by using a Cond 3110 conductivity meter, Germany) and also met the liquid conductivity requirement necessary to enable the OEK chip to work well [36].

### Performance of the separation of Raji cells from RBCs with different concentration ratios

In this study, an electrical field of 20 V_pp_ with frequency of 50 kHz (based on the theoretical calculations as discussed in the ‘Theoretical and FEM modeling’ section) was employed to power the OEK chip. Two optical lines of different widths, i.e., one moving line of 25 μm width and one stationary line of 15 μm width, were adopted as virtual electrodes to generate a DEP force in the OEK chip to separate the Raji cells from the RBCs. Five attempts were done for separating Raji cells from RBCs with two different Raji cells-to RBCs concentration ratios of 1∶10 and 1∶40, respectively. For each time, the Raji cells were successfully manipulated and separated from RBCs.

#### a) A concentration ratio of 1:10

**Figure 8:**
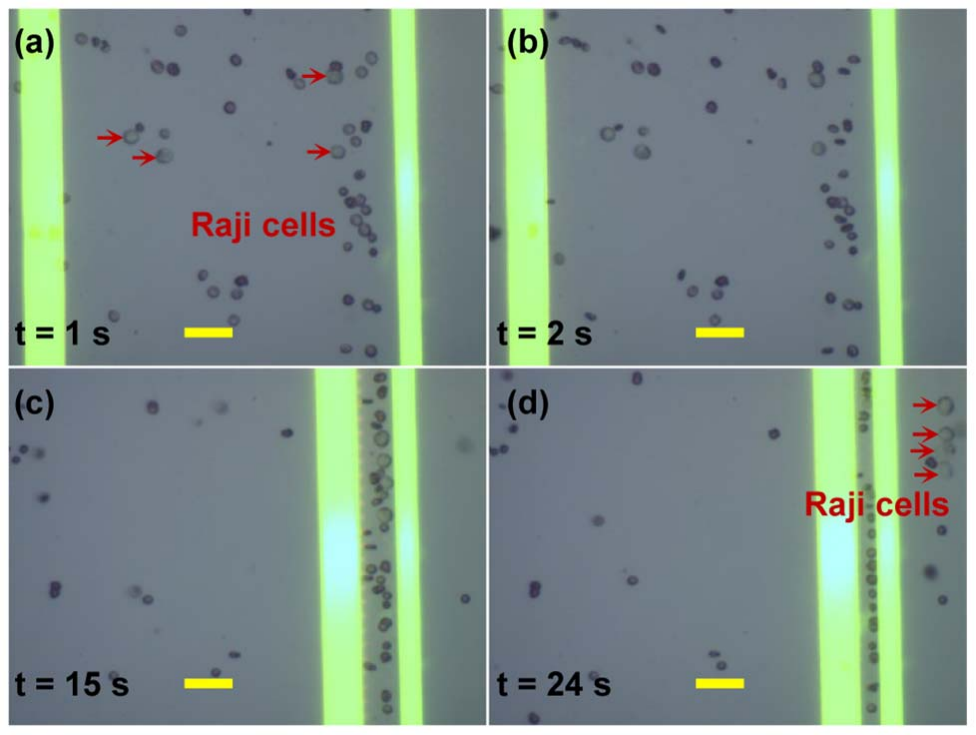
Microscopic images showing the dynamic separation of Raji cells from RBCs with a concentration ratio of 1∶10. All scale bars are 25 μm.

Figure 8 and Video S1 show an example of the experimental process for separating the Raji cells from the RBCs with a concentration of 1∶10 (i.e., approximately 1 Raji cell for every 10 RBCs in the isotonic solution). Initially, when the optical patterns were projected onto the a-Si:H surface and there was no voltage applied, the two types of cells were randomly suspended in the liquid solution as shown in Figure 8(a). Once the voltage was switched on, the two types of cells were repelled, and the RBCs would change their orientations because they would align their longest axis to be parallel to the direction of the electric field (Figure 8(b)). Then, the 25 μm-wide optical line was set with a translational velocity of 12 µm/s from left to right by using an animation software (Flash), and this motion continuously pushed the two types of cells toward the location of the 15 μm optical line, due to the larger nDEP force induced by the 25 μm-wide optical line. We have observed that, when driven by the 25 μm-wide optical line, both of the two types of cells simultaneously have self-rotational and translational behaviors (see Video S1), which are the same observation obtained in our prior experiments with other types of cells as reported in [23]. After 15 s, the two types of cells are aligned and located within the gap between the two lines (Figure 8(c)). When the 25 μm-wide optical line was moved further to the right, all of the Raji cells were pushed past the 15 μm-wide optical line, as shown in Figure 8(d). Whereas, there were also some RBCs pushed towards the stationery line because Raji cells' movement affected those RBCs. The Raji cells could be further separated and purified by employing subsequent virtual electrodes with a similar function explained in this section.

#### b) A concentration ratio of 1∶40

**Figure 9:**
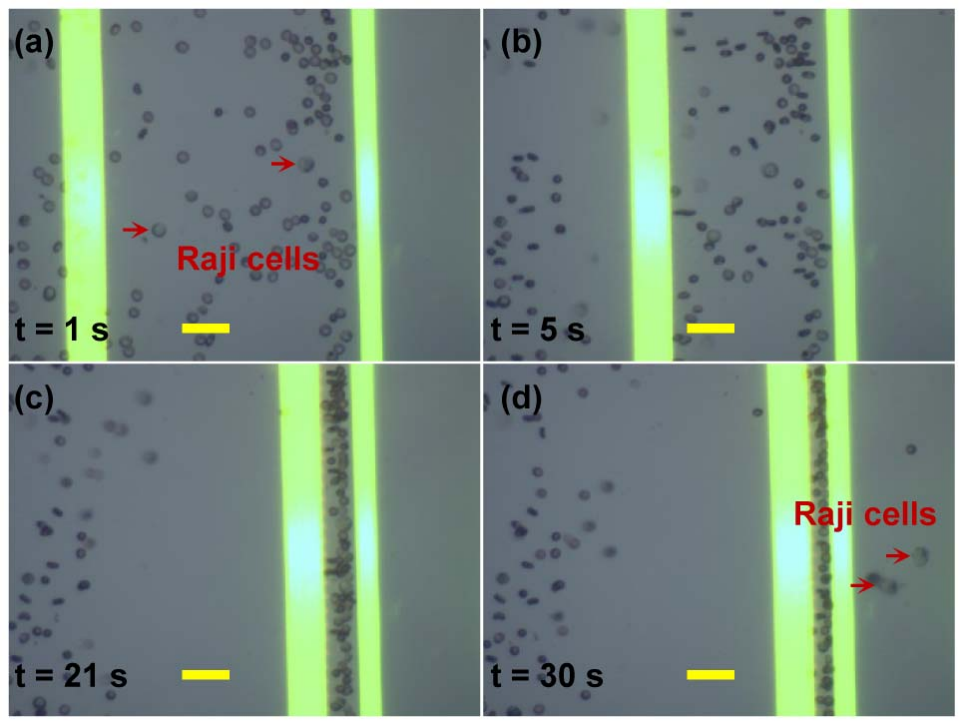
Microscopic images showing the dynamic separation of Raji cells from RBCs with a concentration ratio of 1∶40. All scale bars are 25 μm.

In order to experimentally explore and estimate the throughput of the OEK chip, we further execute the separation experiment of the two types of cells with a lower Raji cells-to-RBCs concentration ratio of 1∶40. Figure 9 and Video S2 present the performance of the separation of Raji cells from the cellular mixture with the same experimental procedure as discussed for the 1∶10 concentration ratio above. As shown in Figure 9(c), there were a few Raji cells and many RBCs trapped within the gap between the two lines. Then, when the gap was further narrowed, the Raji cells were pushed past the stationery line (Figure 9(d)). However, there were some RBCs that were also simultaneously separated. Again, the Raji cells could be further separated and purified from these RBCs by employing subsequent virtual electrodes with a similar function as described above.

The separation efficiency for Raji cells and RBCs of the two different concentrations ratios of 1∶10 and 1∶40 were 67% and 50%, respectively. This efficiency could be easily improved by projecting subsequent virtual electrodes configured as the two optical lines of different widths discussed in this paper to further separate the cells. This process can be repeated in the OEK chip until the cells are completely separated.

The purpose of this study is to verify the capability of using the ODEP technique to separate Raji cells from RBCs, and potentially provide a new automated and label-free technology for cell separation and purification. Since there are much more RBCs in a blood sample than white blood cells, we have focused the current study on only Raji cells and RBCs. We simulated the blood sample of lymphoma patients by spiking the Raji cells (from the cell line described in [34]) into the RBCs sample from volunteers and then perform the cell separation experiment proposed in this study. Although the size of the Raji cells is similar to the white blood cells, theoretically ODEP force can be still applied to discriminate them due to their inherent different dielectric parameters (i.e., membrane/cytoplasm/nucleus permittivity and conductance), which will result in the different direction and/or magnitude of ODEP force exerted on them. We are currently investigating the required electrokinetic parameters for the separation of white blood cells, red blood cells, and Raji cells; we will report our findings in the future.

## Conclusion

We experimentally demonstrated rapid separation of Raji cells from red blood cells (RBCs) by employing dynamic *optically-induced dielectrophoretic* (ODEP) force on the cells. The separation of these cells with two different concentration ratios was achieved by using two optically-projected lines (one dynamically moving and one fixed) that generate negative ODEP force of different magnitudes on the cells. Inside an *optically-induced electrokinetics* (OEK) chip, these projected line images act as virtual electrodes to generate the negative ODEP force on the cells under a non-uniform electric field produced by a 20 V_pp_ at frequency of 50 kHz across the fluidic medium. We also report the selection of an appropriate isotonic solution with the purpose of addressing the problems of cellular adhesion behavior and proper fluid conductivity for cell separation in an OEK chip. In addition, an FEM simulation of the ODEP force acting on the cells was carried out to explain the separation phenomenon. This simulation includes the usage of different polarization models for the Raji cells and RBCs. Further work is required to investigate the viability of cells using the separation technique reported in this paper. However, our initial work showed that both Raji cells and RBCs do survive for several hours after the separation experiments in an OEK chip. Pending on the results of more extensive cell viability experiments, cell separation using dynamic ODEP forces could prove to be a unique method that is capable of rapidly separating and purifying cells using relative simple procedures.

## Supporting Information

Figure S1 **A picture of the actual ODEP system setup used to manipulate and separate cells in our experiments.**
(TIF)

Video S1 **Video of the experimental results of the dynamic separation of Raji cells from RBCs with a concentration ratio of 1∶10.**
(AVI)

Video S2 **Video of the experimental results of the dynamic separation of Raji cells from RBCs with a concentration ratio of 1∶40.**
(AVI)
