# Supplementary figures and images for: Rapid and Label-Free Separation of Burkitt's Lymphoma Cells from Red Blood Cells by Optically-Induced Electrokinetics
Source: PLoS One. 2014 Mar 7;9(3):e90827. doi: 10.1371/journal.pone.0090827 (PMC3946566; doi:10.1371/journal.pone.0090827)

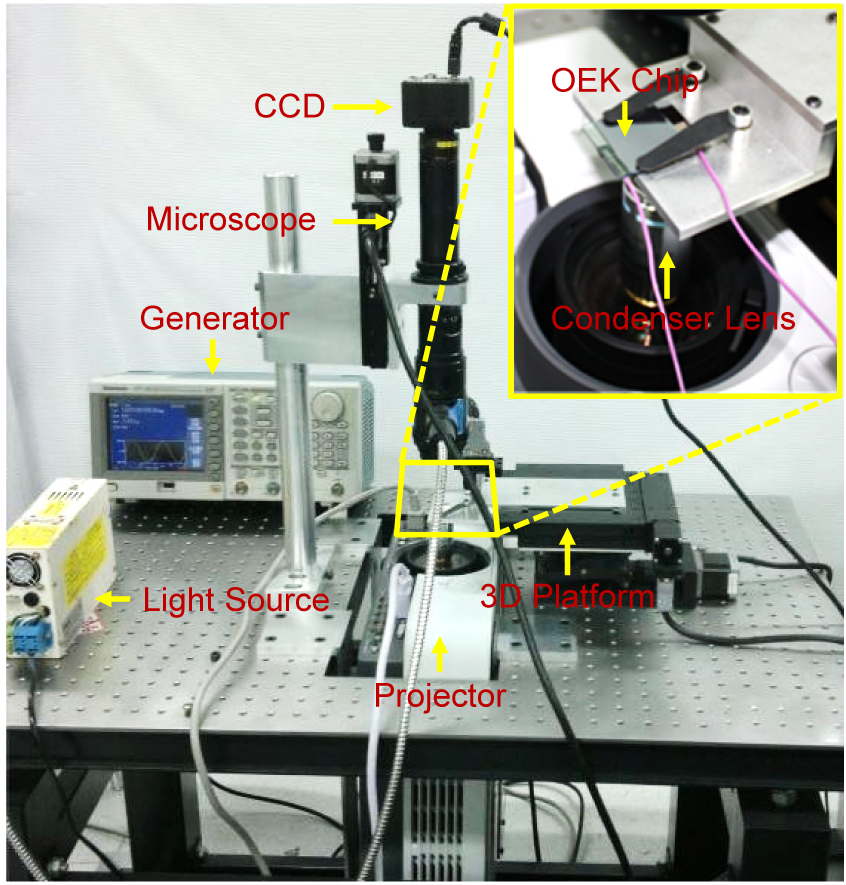

Supplement: Figure S1 — A picture of the actual ODEP system setup used to manipulate and separate cells in our experiments. (TIF) [file pone.0090827.s001.tif]
